# Supplementary material for: The β2-adrenergic biased agonist nebivolol inhibits the development of Th17 and the response of memory Th17 cells in an NF-κB-dependent manner
Source: Front Immunol. 2024 Oct 9;15:1446424. doi: 10.3389/fimmu.2024.1446424 (PMC11496295; doi:10.3389/fimmu.2024.1446424)
Supplement: Supplementary file 1 [file DataSheet1.docx]

# **Supplementary Data**


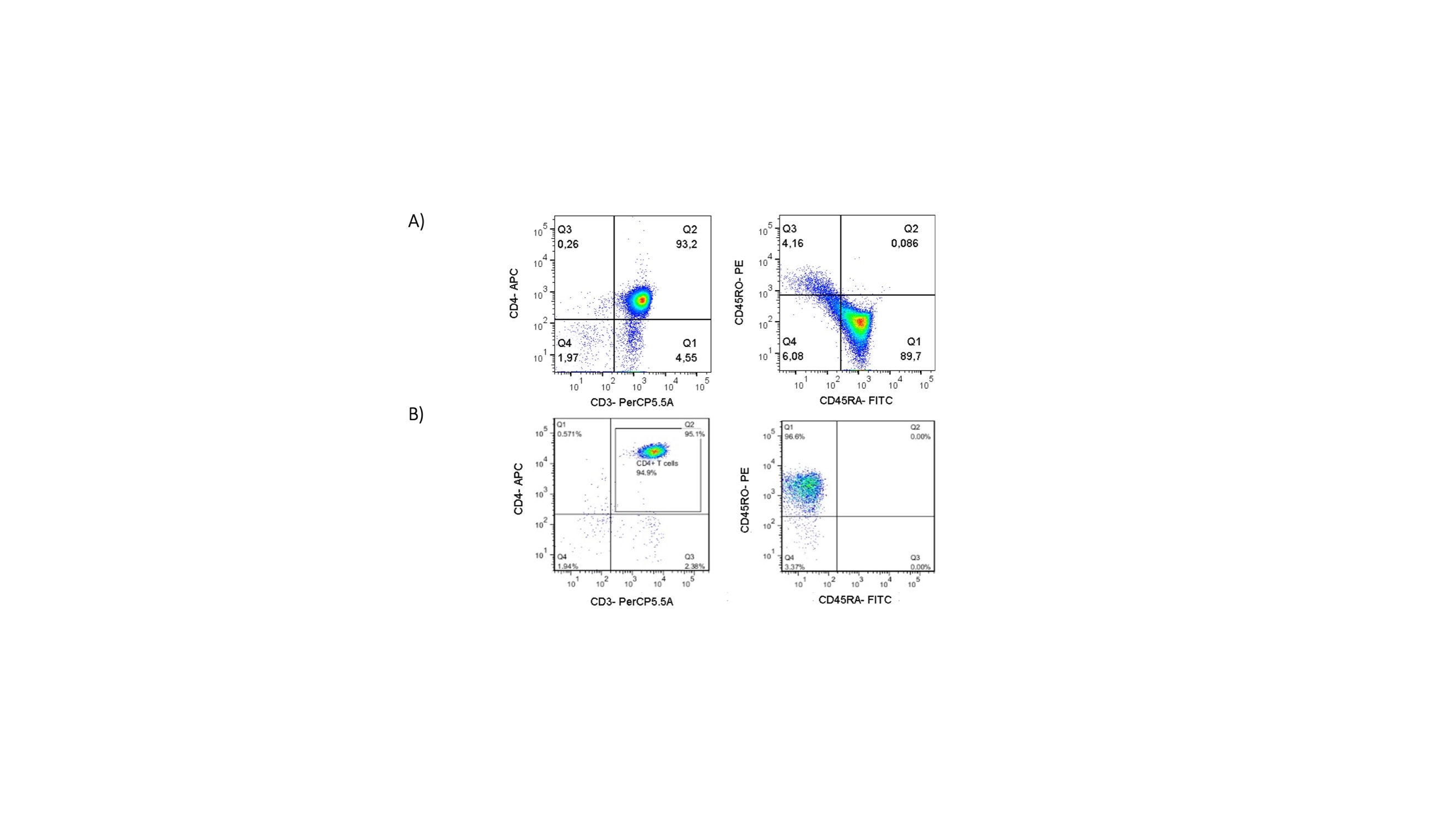


**Supplementary Figure 1.** Purification checks of A) naïve and B) memory Th cells after using EasySep® naïve and memory CD4^+^ T cell enrichment kits respectively.


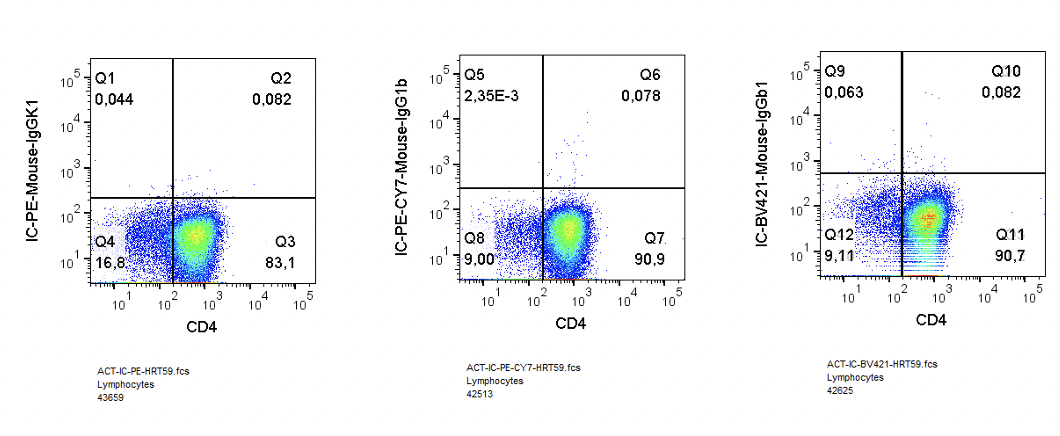
**Supplementary Figure 2.** Isotype controls the results of intracellular cytokine staining (ICS).


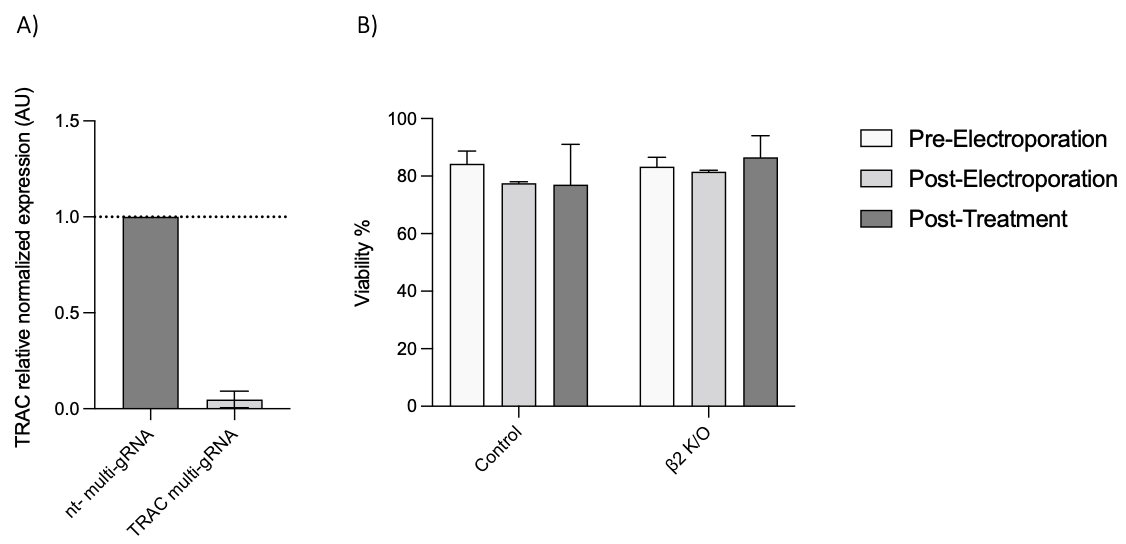


**Supplementary Figure 3.** A) Verification of CRISPR/Cas9 knockouts of the TRAC gene as a positive control in memory Th cells at the mRNA level. The graph includes non-targeting (nt) and TRAC multi-sgRNA conditions. Results are shown as relative amounts normalized to housekeeping RNA, compared to nt-multi-gRNA, set to 1.0 (dotted line). B) Viability assessment of non-electroporated and ADRB2 multi-sgRNA conditions before and after electroporation on day 4 and after 5 days of treatment. Data represent the mean ± SEM from two experiments. Statistical analysis was performed using one-way ANOVA followed by Tukey’s multiple comparisons test.


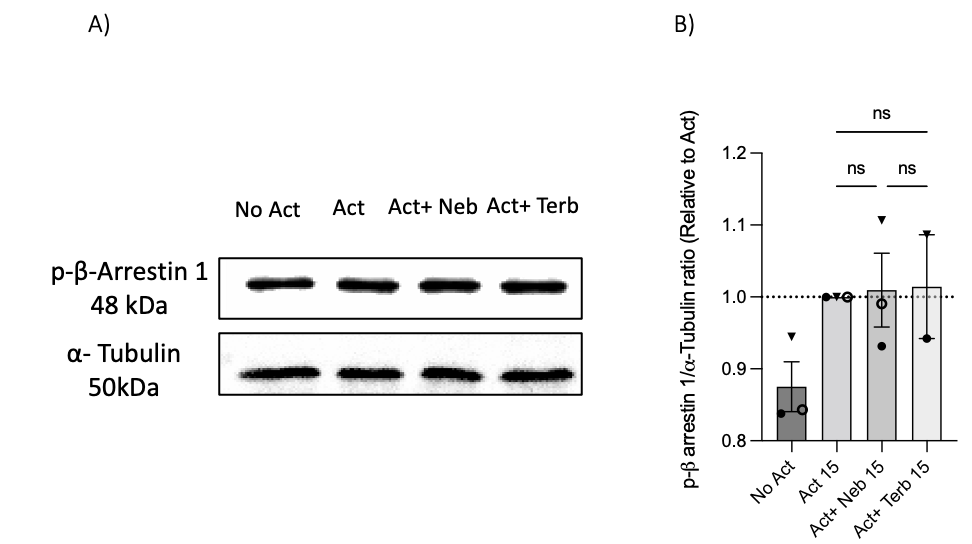


**Supplementary Figure 4.** Nebivolol did not stimulate phospho-beta-arrestin-1 (p-ß Arrestin1) in memory Th cells. Blasted memory Th cells were activated with ImmunoCult for 15 mins in conditions of non-activated cells, activated cells, activated cells plus nebivolol, or activated cells plus terbutaline A) Representative western blot data of equal amounts of protein from the cell lysates was shown for p-ß arrestin1 and α-tubulin as a loading control. Band intensity was quantified and shown corrected to the loading control, and relative to the non-activation, which was set to 1 for p-ß arrestin1 (dotted line), with data pooled from 3 experiments.

**Supplementary Table 1,** payload sequence of multi-guide RNA for targeting TRAC and ADRB2 genes.

| Human TRAC multi-guide sgRNA, modified | sgRNA 1: 5'-CUCUCAGCUGGUACACGGCA-3' |
| --- | --- |
|  | sgRNA 2: 5'-GAGAAUCAAAAUCGGUGAAU-3' s |
|  | gRNA 3: 5'-ACAAAACUGUGCUAGACAUG-3' |
| Human ADRB2 multi-guide sgRNA, modified | sgRNA 1: 5'-GCCGUUCCCGGGUUGCCCCA-3' |
|  | sgRNA 2: 5'-ACCACGACGUCACGCAGGAA -3' |
|  | gRNA 3: 5'-CGUCAUGUCUCUCAUCGUCC -3' |
